# POSTOPERATIVE OUTCOME OF PATIENTS ADMITTED TO THE INTENSIVE CARE UNIT AFTER ELECTIVE AND EMERGENCY LAPAROTOMY

**DOI:** 10.1590/0102-67202025000010e1879

**Published:** 2025-04-25

**Authors:** Murilo Tavares VALVERDE, Gabriel Vianna Pereira ARAGÃO, Igor Lima Vieira de CASTRO, Jade de Oliveira SANTANA, Liana CODES, Claudio Celestino ZOLLINGER, Wellington ANDRAUS, Paulo Lisboa BITTENCOURT

**Affiliations:** 1 Escola Bahiana de Medicina e Saúde Pública, Department of Gastroenterology - Salvador (BA), Brazil; 2 Hospital Português, Unit of Gastroenterology and Hepatology - Salvador (BA), Brazil; 3 Universidade de São Paulo, Department of Gastroenterology - São Paulo (SP), Brazil

**Keywords:** Morbidity, Mortality, General Surgery, Intensive Care Units, Morbidade, Mortalidade, Cirurgia Geral, Unidades de Terapia Intensiva

## Abstract

**BACKGROUND:**

Surgery is associated with a high risk for morbidity and mortality, particularly when performed in critical patients requiring intensive care unit (ICU) admission.

**AIM:**

The aim of this study was to investigate risk factors associated with adverse outcomes in a large cohort of patients admitted to a single-center ICU after abdominal surgery.

**METHODS:**

All patients admitted to a surgical ICU for postoperative care, from January 2016 to December 2022, were retrospectively evaluated. Data concerning demographics and clinical and perioperative variables were compared to in-hospital mortality.

**RESULTS:**

A total of 1,717 patients (1,096 women, mean age: 61±17 years) were evaluated. Most of the patients underwent colorectal (n=499), pancreatic (n=148), biliary tract (n=147), and gastric surgeries (n=145); liver resection (n=131); and several gynecological or obstetric procedures (n=250). Only 52.3% of these surgical procedures were elective. The mean Charlson Comorbidity Index (CCI) and Acute Physiology and Chronic Health Evaluation II (APACHE II) scores were 4.4±2.8 and 10.1±5.6, respectively. Mortality was observed in 158 (9.2%) patients. Age (70.4±14.3 vs. 60.6±17.1 years in survivors, p=0.002), CCI (6.1±2.5 vs. 4.3±2.8 in survivors, p=0.005), type of surgery (13.6% in emergent/urgent vs. 5.5% in elective surgeries, p<0.001), and APACHE II score (16.7±8.4 vs. 9.4±4.7 in survivors, p<0.0001) were associated with mortality on univariate analysis, but only CCI, type of surgery, and APACHE II score were independently correlated with a higher risk of death on multivariate analysis.

**CONCLUSIONS:**

Mortality after abdominal surgery in patients requiring postoperative ICU support is less than 10% nowadays, and it is independently associated with urgent or emergent surgeries, disease severity, and comorbidity.

## RESUMO

**RACIONAL:**

Cirurgias estão associadas a um alto risco de morbidade e mortalidade quando realizadas em pacientes críticos.

**OBJETIVOS:**

investigar os fatores de risco associados a resultados adversos em uma coorte de pacientes admitidos em unidade de terapia intensiva (UTI) de um único centro de referência após cirurgia abdominal.

**MÉTODOS:**

Pacientes admitidos em UTI cirúrgica entre janeiro 2016 e dezembro 2022 foram avaliados retrospectivamente. Dados referentes a variáveis demográficas, clínicas e perioperatórias foram comparados à mortalidade hospitalar.

**RESULTADOS:**

Foram avaliados 1.717 pacientes (1.096 mulheres, idade 61+17 anos). A maioria dos pacientes foi submetida a cirurgia colorretal (n=499), pancreática (n=148), biliar (n=147), gástrica (n=145), hepática (n=131) e vários procedimentos ginecológicos/obstétricos (n=250). Um total de 52,3% desses procedimentos cirúrgicos foram eletivos. Os escores médios do Índice de Comorbidade de Charlson (CCI) e do APACHE II foram 4,4±2,8 e 10,1±5,6, respectivamente. Mortalidade foi observada em 158 (9,2%) pacientes. Idade (70,4±14,3 vs. 60,6±17,1 anos nos sobreviventes, p=0,002), CCI (6,1±2,5 vs. 4,3±2,8 nos sobreviventes, p=0,005), tipo de cirurgia (13,6% cirurgias emergenciais ou urgentes vs. 5,5% cirurgias eletivas, p<0,001) e APACHE II (16,7±8,4 vs. 9,4±4,7 nos sobreviventes, p=0,001) foram associados à mortalidade na análise univariada, mas somente o índice de comorbidade de Charlson, o tipo de cirurgia e APACHE II foram independentemente correlacionados a um maior risco de morte na análise multivariada.

**CONCLUSÕES:**

Atualmente, a mortalidade após cirurgia abdominal em pacientes que necessitam de suporte pós-operatório em UTI é inferior a 10%, sendo independentemente associada a cirurgias urgentes/emergenciais, gravidade da doença e comorbidades.

**Figure:**
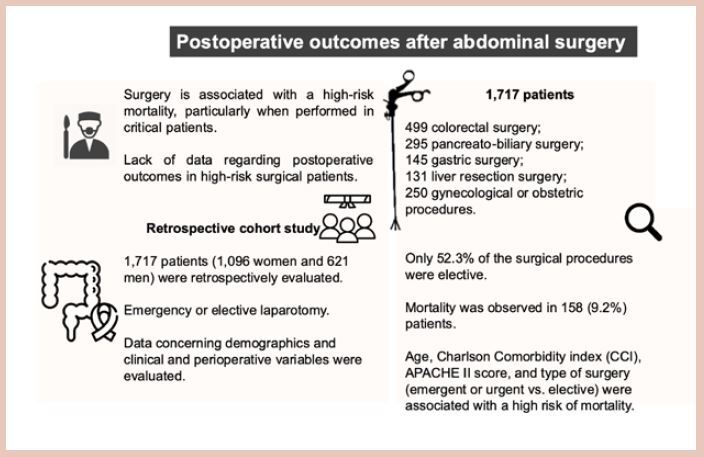
Outcomes of surgical patients admitted to the intensive care unit after elective and emergency surgeries in a tertiary care hospital in Brazil.

**Central Message**

In Brazil, data related to the prognosis of surgical patients are scarce. We sought to assess the outcomes of surgical patients admitted to an intensive care unit (ICU) following elective and urgent procedures in a tertiary care hospital in Brazil, as well as to examine risk factors related to mortality. For this purpose, a retrospective study was carried out, and a total of 1,717 patients were evaluated. Mortality was observed in 158 (9.2%) patients, and it was associated with urgent or emergent surgeries, disease severity, and comorbidities.

**Perspectives**

Knowledge of factors that influence the prognosis of surgical patients is useful because not only we can make adequate risk stratification but also it can help develop strategies to improve the outcomes of this group of patients.

## INTRODUCTION

Outcomes after gastrointestinal and hepatobiliary surgeries were shown to vary sharply according to age and comorbidity^4^ ^,^ ^5^ ^,^ ^14^ ^,^ ^18^, type of surgery^2^ ^,^ ^7^, surgeon’s technical skills and experience^3^ ^,^ ^8^ ^,^ ^17^ ^,^ ^20^, and quality of postoperative care with respect to human resources and hospital infrastructure^13^ ^,^ ^22^. Emergency laparotomy has been associated with increased mortality, but better outcomes after emergency laparotomy have been related to the availability of intensive care unit (ICU) or high-dependency beds in some studies^13^ ^,^ ^15^ ^,^ ^22^.

Mortality is usually ascribed to the development of surgical complications leading to sepsis and multi-organ failure^9^. There are only few studies in Brazil concerning the outcome of emergency or elective laparotomy in high-risk patients admitted to the ICU for postoperative care^9^ ^,^ ^10^ ^,^ ^18^. Nowadays, postoperative deaths are the third most common cause of mortality worldwide^11^, and the investigation of major perioperative risk factors associated with worse outcomes is crucial to decrease surgical morbidity and mortality^1^.

The purpose of the present study was to evaluate the outcomes of surgical patients admitted to a single-center ICU after elective and emergency surgeries in a tertiary care hospital in Brazil, as well as to investigate risk factors associated with in-hospital mortality.

## METHODS

All patients admitted to the Gastroenterology and Hepatology Unit of Hospital Português, Salvador (BA), Brazil, after elective or emergency laparotomy, from January 2016 to December 2022, were retrospectively evaluated except for those patients admitted after organ transplantation. This facility is an intensive gastrointestinal ICU dedicated to the postoperative care of high-risk patients submitted to abdominal surgery.

Data concerning demographics; year at admission; type and duration of surgery; surgical procedure; surgical wound classification; surgical team; comorbidity, according to Charlson Comorbidity Index (CCI) and presence of concurrent malignancy and Acute Physiology and Chronic Health Evaluation II (APACHE II) score, in the first 24 h in the ICU; ICU stay and intrahospital length of stay (LOS); and mortality were retrospectively reviewed. Patients in palliative care were excluded from the analysis. Surgery was considered elective when it was scheduled or planned and urgent in the presence of an acute event leading to admission in the emergency department and requiring surgery in the first 24 h. The immediate need for surgery, due to potentially fatal diseases, was the justification for indicating emergency surgical interventions. Surgical wound grades, as well as APACHE II score and CCI, were classified and calculated as previously described^5^ ^,^ ^16^ ^,^ ^21^.

Surgical teams were arbitrarily defined by a group of certified surgeons and surgeons’ assistants as well as surgical technologists usually working together in the surgical theater. In our institution, surgeons and anesthesiologists are third-party, and surgical nurses are part of hospital staff. Surgical teams were labeled for anonymization purposes, as letters A-Q. Surgical teams who performed less than 25 surgeries in the observation period were grouped in label Q. Surgical procedures were grouped as appendicectomy, bariatric, colorectal, cytoreductive, enterectomy, esophagectomy, gastrectomy, gynecologic or obstetric, liver resection, pancreatic, biliary tract, retroperitoneal, splenectomy, and urologic surgeries. Emergent laparotomy was considered in emergent surgeries when no organ resection was carried out in the presence, for example, of adhesions or hernia repair. Patients were followed up until death or hospital discharge. The primary endpoint was in-hospital mortality.

The study was approved by the Ethics Committee in Research of the Portuguese Hospital Portugues, Salvador (BA), Brazil (number: 26210819.5.0000.5029).

### Statistical analysis

Dichotomous variables were presented in text and tables as numbers and percentages, and continuous variables were expressed as mean±standard deviation (SD) or as median and interquartile range, respectively, based on whether the distribution was normal or skewed. Data concerning surgical procedures were compared using the chi-square test or Fisher’s exact test for categorical variables or Student’s t-test or the Mann-Whitney U test for continuous variables when appropriate. Variables associated with mortality in univariate analysis with a p<0.10 were entered in a multivariate logistic regression model using stepwise elimination. Year at admission was also considered in the multivariate logistic regression model independent of p-values to assess a possible effect of the COVID-19 pandemic on surgical outcomes. A p<0.05 was considered statistically significant. The software used for the analysis was the Statistical Package for Social Sciences (SPSS Inc., Chicago, IL, USA), version 14.0 for Windows.

## RESULTS

**Figure 1:**
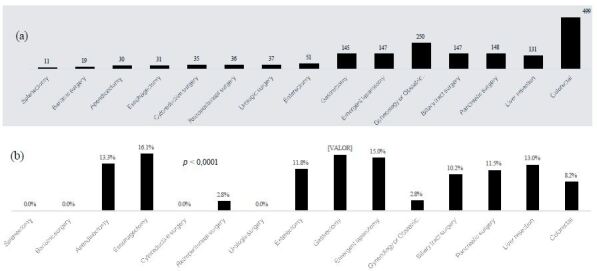
(a) Number of surgical procedures leading to ICU admission, (b) postoperative mortality according to surgical procedures (n=1,717).

A total of 1,717 consecutive patients (1,096 women, mean age: 61±17 years) were admitted to the ICU after surgery. Demographics and clinical and postoperative features of the patients are summarized in Table 1. Most of the patients underwent colorectal (n=499), pancreatic (n=148), biliary tract (n=147), and gastric (n=145) surgeries; liver resection (n=131); emergent laparotomy (n=147); and several gynecological or obstetric procedures (n=250) (Figure 1a). The median CCI was 4.4±2.8, and 62% of the patients had concurrent malignancy. According to type, 52.8% of the surgical procedures were elective, 45.9% were urgent, and only 1.3% were emergent. The mean duration of surgery was 3.9±2.1 h and according to the CDC classification, most surgical wounds were clean-contaminated (Table 1). The mean postoperative APACHE II score in the first 24 h of ICU admission was 10.1±5.6. A total of 158 (9.2%) patients died after a mean ICU stay and in-hospital LOS of 5.7 [2-6] and 11.9 [4-14] days, respectively.

**Table 1:** Demographics and clinical and postoperative features of surgical patients.

Features	n=1,717
**Age (years)**	61±17
**Gender**	
Male	621 (36%)
Female	1,096 (34%)
**Comorbidity**	
CCI (mean)	4.4±2.8
Cancer	1,069 (62%)
**Type of surgery**	
Elective	907 (52.8%)
Urgent	788 (45.9%)
Emergent	22 (1.3%)
**Wound classification**	
Clean	274 (16%)
Clean-contaminated	1,171 (68.2%)
Contaminated	163 (9.5%)
Dirty/infected	109 (6.3%)
**Surgery duration (hours)**	3.9±2.1
**Outcomes**	
Postoperative APACHE II	10.4±5.7
ICU LOS (days)	5.7 [2-6]
Hospital LOS (days)	11.9 [4-14]
Mortality	158 (9.2%)

ICU: intensive care unit; LOS: length of stay; CCI: Charlson Comorbidity Index; APACHE II: Acute Physiology and Chronic Health Evaluation II.

**Figure 2:**
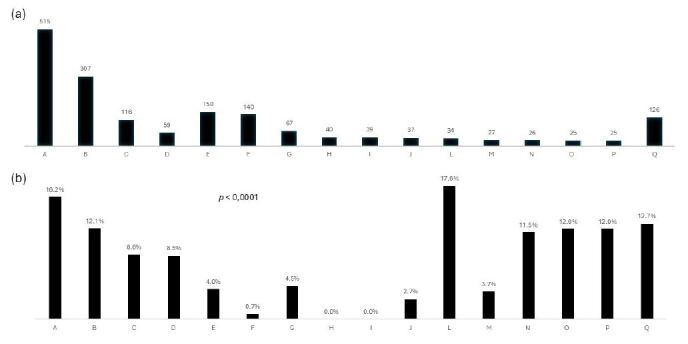
(a) Number and volume of surgical teams, (b) postoperative mortality according to surgical teams (n=1,717).

Mortality was significantly different according to the type of surgical procedure and surgical teams (Figures 1 and 2). In this regard, esophagectomy (16.1%), gastrectomy (15.9%), and emergent laparotomy (15%) had the highest mortality rates (Figure 1b).

Comparison of the demographics and clinical and postoperative features of the patients according to mortality revealed that age, male gender, comorbidity assessed by the CCI, type of surgery, wound classification, surgery duration, and postoperative APACHE II scores were significantly associated with mortality. Conversely, subjects who died had significantly prolonged ICU stay and in-hospital LOS (Table 2).

**Table 2:** Demographics and clinical and postoperative features of surgical patients according to mortality.

	Patients discharged alive (n=1,559)	Dead patients (n=158)	p-value
Age (years)	60.6±17.1	70.4±14.3	0.002
Gender			0.045
Male	552 (88.9%)	69 (11.1%)	
Female	1007(91.9%)	89 (8.1%)	
Comorbidity			
CCI (mean)	4.3±2.8	6.1±2.5	0.005
Cancer	963 (90.1)	106 (9.9)	0.109
Type of surgery			<0.0001
Elective	857 (94.5%)	50 (5.5%)	
Urgent	683 (86.7%)	105 (13.3%)	
Emergency	19 (86.4%)	3 (13.6%)	
Wound classification			0.001
Clean	258 (94.2%)	16 (5.8%)	
Clean-contaminated	1069 (91.3%)	102 (8.7%)	
Contaminated	142 (87.1%)	21 (12.9%)	
Dirty/infected	90 (82.6%)	19 (17.4%)	
Surgery duration (hours)	3.9±2.0	4.2±2.5	<0.0001
Outcomes			
Postoperative APACHE II	9.4±4.7	16.7±8.4	<0.0001
ICU LOS (days)	5.2±6.4	10.7±12.5	<0.0001
Hospital LOS (days)	11.4±14.3	16.9±22.8	<0.0001

APACHE II: Acute Physiology and Chronic Health Evaluation II; ICU: intensive care unit; LOS: length of stay; CCI: Charlson Comorbidity Index.

On univariate analysis, mortality was associated with male gender (odds ratio [OR]=1.41; 95% confidence interval [CI] 1.02-1.97; p=0.04), age (OR=1.04; 95%CI 1.03-1.05, p<0.0001), type of surgical procedure (OR=2.63; 95%CI 1.85-3.74; p<0.0001), APACHE II score (OR=1.20; 95%CI 1.17-1.23; p<0.0001), CCI (OR=1.24; 95%CI 1.17-1.32; p<0.0001), and hospital stay and ICU LOS (OR=1.015; 95%CI 1.007-1.023; p<0.0001 and OR=1.063; 95%CI 1.05-1.08; p<0.0001, respectively). Only the type of surgery (OR=2.48; 95%CI 1.66-3.71; p<0.0001), APACHE II score (OR=1.18; 95%CI 1.15-1.22; p=0.0001), CCI (OR=1.16; 95%CI 1.07-1.26; p=0.004), and ICU LOS (OR=1.08; 95%CI 1.05-1.11; p=0.001) remained statistically significant on multivariate analysis. Surgical teams and procedures as well as year at admission were not associated with mortality on multivariate analysis (Table 3).

**Table 3:** Univariate and multivariate analyses of variables associated with mortality in subjects admitted to the ICU after surgery.

Variables	Univariate analysis			Multivariate analysis		
	OR	95%CI	p-value	OR	95%CI	p-value
Gender	1.41	1.02-1.97	0.04			
Age	1.04	1.03-1.05	0.002			
Type of surgery	2.63	1.85-3.74	<0.0001	2.48	1.66-3.71	<0.0001
CCI	1.24	1.17-1.32	0.005	1.16	1.07-1.26	0.004
APACHE II	1.20	1.17-1.23	<0.0001	1.18	1.15-1.22	0.0001
ICU LOS	1.063	1.05-1.08	<0.0001	1.08	1.05-1.11	0.0001
Hospital LOS	1.015	1.007-1.023	<0.0001			

APACHE II: Acute Physiology and Chronic Health Evaluation II; CCI: Charlson Comorbidity Index; ICU: intensive care unit; LOS: length of stay; CI: confidence interval.

## DISCUSSION

The present study showed that 9.2% of the patients admitted to the ICU after abdominal surgery died and the mortality was independently associated with emergent or urgent surgery, disease severity, and comorbidity assessed, respectively, by APACHE II score and CCI. Our data are in accordance with previous reports showing mortality rates for high-risk surgical patients admitted to the ICU ranging from 9.6% to 19%^7^ ^,^ ^17^, particularly after either emergent or urgent^13^ ^,^ ^19^ ^,^ ^23^ or abdominal surgery^15^. One of those studies was performed in Brazil^18^ involving 904 high-risk patients, admitted to 55 different ICUs after non-cardiac surgery. The 28-day morbidity and mortality rates were, respectively, 29.9 and 9.6%. In contrast to our study, most of those procedures were elective and only one-third of them were abdominal surgeries^18^. In this study, disease severity and organ dysfunction at admission, assessed by SAPS III (Simplified Acute Physiology Score III) and SOFA (Sequential Organ Failure Assessment) scores, and older age were also associated with worse outcomes after surgery. In our larger cohort, older age, gender, and duration of surgery were also associated with mortality in univariate but not in multivariate analysis. In addition, as previously highlighted in several reports^6^ ^,^ ^15^, comorbidity was independently associated with mortality. Since that not all elective patients go to the ICU after surgery, just the ones with severe comorbidities or those who underwent more complex surgeries, we can infer that the difference between urgent and elective surgical patients could be even higher than the one observed in the present study^2^.

Pearse et al.^15^ reported a 7-day cohort study across Europe that evaluated non-cardiac surgical outcomes in more than 45,000 patients. Only 8% were critically ill patients requiring postoperative ICU admission. Mortality before hospital discharge was 4%, but even in these less severe subjects, emergent or urgent procedures, gastrointestinal or hepatobiliary surgeries, and several comorbidities were associated with an increased risk for mortality^15^. It is interesting to point out that mortality was shown to vary according to surgical teams and surgical procedures, but these variables were not independently associated with mortality after surgery. The surgical teams were dedicated to different surgical procedures that may have different outcomes and complication rates as seen in our results. This fact justified the difference in their results and mortality. Patients who died had longer ICU stays, indicating a higher use of healthcare resources and a higher cost in an attempt to overcome surgical complications^12^.

The knowledge of the type of procedures that have more complications and mortality is very important in ICU care and management. Having that in mind, we can search, anticipate, and treat patients with higher chances of a worse outcome.

## CONCLUSIONS

Mortality after abdominal surgery in patients admitted to the ICU, in the largest single-center Brazilian cohort investigated this far, was below 10%. Only emergent or urgent surgery, disease severity, and comorbidity were independently associated with an increased risk of death.

## References

[1] Almeida GF, Silva PPCE, Valverde MT, Morais MCA, Chagas PBO, D'Oliveira RAC (2023). Acute abdomen in intensive care unit: etiology, comorbidity and severity of 1,523 patients. Arq Bras Cir Dig.

[2] Birkmeyer JD, Siewers AE, Finlayson EV, Stukel TA, Lucas FL, Batista I (2002). Hospital volume and surgical mortality in the United States. N Engl J Med.

[3] Boyd-Carson H, Doleman B, Herrod PJJ, Anderson ID, Williams JP, Lund JN (2019). Association between surgeon special interest and mortality after emergency laparotomy. Br J Surg.

[4] Charlson ME, Pompei P, Ales KL, MacKenzie CR (1987). A new method of classifying prognostic comorbidity in longitudinal studies: development and validation. J ChronicDis.

[5] Clarke A, Murdoch H, Thomas MJ, Cook TM, Peden CJ (2011). Mortality and postoperative care after emergency laparotomy. Eur J Anaesthesiol.

[6] Clements NA, Gaskins JT, Martin RCG (2023). Predictive ability of comorbidity indices for surgical morbidity and mortality: a systematic review and meta-analysis. J GastrointestSurg.

[7] Dimick JB, Staiger DO, Birkmeyer JD (2006). Are mortality rates for different operations related? Implications for measuring the quality of noncardiac surgery. MedCare.

[8] Gillies MA, Power GS, Harrison DA, Fleming A, Cook B, Walsh TS (2015). Regional variation in critical care provision and outcome after high-risk surgery. Intensive Care Med.

[9] Lobo SM, Rezende E, Knibel MF, Silva NB, Páramo JA, Nácul FE (2011). Early determinants of death due to multiple organ failure after noncardiac surgery in high-risk patients. Anesth Analg.

[10] Lobo SM, Rezende E, Knibel MF, Silva NB, Páramo JA, Nácul F (2008). Epidemiology and outcomes of non-cardiac surgical patients in Brazilian intensive care units. Rev Bras Ter Intensiva.

[11] Nepogodiev D, Martin J, Biccard B, Makupe A, Bhangu A, National Institute for Health Research Global Health Research Unit on Global Surgery (2019). Global burden of postoperative death. Lancet.

[12] Oliveira RCC, Malafaia O, Tabushi FI, Naufel CR, Lourenco ES, Tabushi FY (2022). Intensive care unit prescriptions must fit risk factors to prevent stress ulcer bleeding. Arq Bras Cir Dig.

[13] Ozdemir BA, Sinha S, Karthikesalingam A, Poloniecki JD, Pearse RM, Grocott MP (2016). Mortality of emergency general surgical patients and associations with hospital structures and processes. Br J Anaesth.

[14] Pearse RM, Harrison DA, James P, Watson D, Hinds C, Rhodes A (2006). Identification and characterisation of the high-risk surgical population in the United Kingdom. Crit Care.

[15] Pearse RM, Moreno RP, Bauer P, Pelosi P, Metnitz P, Spies C (2012). Mortality after surgery in Europe: a 7 day cohort study. Lancet.

[16] Salluh JI, Soares M (2014). ICU severity of illness scores: APACHE, SAPS and MPM. Curr Opin Crit Care.

[17] Schuster KM, Hazelton JP, Rattigan D, Perez JM, Bhattacharya B (2021). Association of acute care surgeon experience with emergency surgery patient outcomes and mortality. JAMA Surg.

[18] Silva JM, Chaves RCF, Corrêa TD, Assunção MSC, Katayama HT, Bosso FE (2020). Epidemiology and outcome of high-surgical-risk patients admitted to an intensive care unit in Brazil. Rev Bras Ter Intensiva.

[19] Stahlschmidt A, Novelo B, Alexi Freitas L, Cavalcante Passos S, Dussán-Sarria JA, Félix EA (2018). Predictors of in-hospital mortality in patients undergoing elective surgery in a university hospital: a prospective cohort. Braz J Anesthesiol.

[20] Stulberg JJ, Huang R, Kreutzer L, Ban K, Champagne BJ, Steele SR (2020). Association between surgeon technical skills and patient outcomes. JAMA Surg.

[21] (2015). National Healthcare Safety Network. Surgical Site Infection (SSI).

[22] Symons NR, Moorthy K, Almoudaris AM, Bottle A, Aylin P, Vincent CA (2013). Mortality in high-risk emergency general surgical admissions. Br J Surg.

[23] Vester-Andersen M, Lundstrøm LH, Møller MH, Waldau T, Rosenberg J, Møller AM (2014). Mortality and postoperative care pathways after emergency gastrointestinal surgery in 2904 patients: a population-based cohort study. Br J Anaesth.

