# ERRATUM

**DOI:** 10.1590/0102-67202025000010e1879ERRATUM

**Published:** 2025-06-27

**ERRATUM:** Postoperative outcome of patients admitted to the intensive care unit after elective and emergency laparotomy

In the manuscript "Postoperative outcome of patients admitted to the intensive care unit after elective and emergency laparotomy", https://doi.org/10.1590/0102-67202025000010e1879, published in the Arq. Bras. Cir. Dig.. 2025;38:e1879, on page 3:

**Where it reads:**

**Figure 1:**
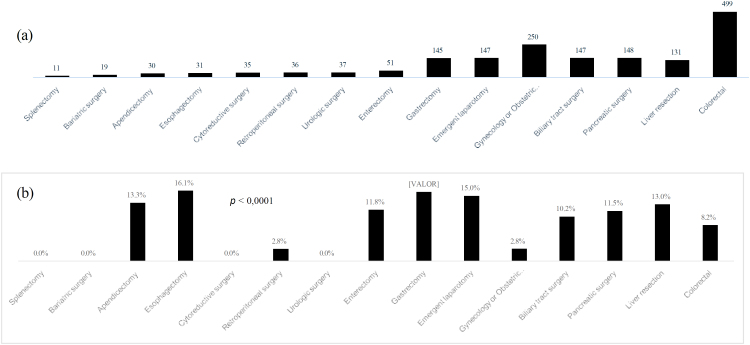
(a) Number of surgical procedures leading to ICU admission, (b) postoperative mortality according to surgical procedures (n=1,717).

**It should read:**

**Figure 1:**
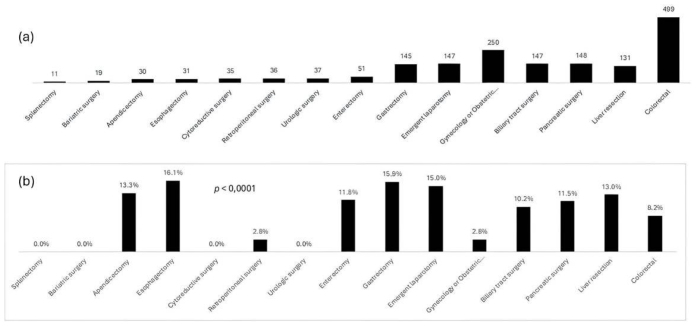
(a) Number of surgical procedures leading to ICU admission, (b) postoperative mortality according to surgical procedures (n=1717).

